# Isocyanate- and solvent-free synthesis of melt processible polyurea elastomers derived from urea as a monomer†

**DOI:** 10.1039/d0ra02369h

**Published:** 2020-05-18

**Authors:** B. Tyler White, John M. Migliore, Emmanuel U. Mapesa, Josh D. Wolfgang, Joshua Sangoro, Timothy E. Long

**Affiliations:** Department of Chemistry, Macromolecules Innovation Institute (MII), Virginia Tech Blacksburg VA 24061 USA telong@vt.edu +1 540 231 8517 +1 540 231 2480; Department of Chemistry, Bethel University St. Paul MN 55112 USA; Department of Chemical and Biomolecular Engineering, University of Tennessee Knoxville TN 37996 USA

## Abstract

Polyurea elastomers are utilized for a myriad of applications ranging from coatings and foams to dielectric materials for capacitors and actuators. However, current synthetic methods for polyureas rely on highly reactive isocyanates, solvents, and catalysts, which collectively pose serious safety considerations. This report details the synthesis and characterization of melt processible, poly(tetramethylene oxide) (PTMO)-based segmented polyurea elastomers utilizing an isocyanate-, solvent-, and catalyst-free approach. Dynamic mechanical analysis and differential scanning calorimetry suggested microphase separation between the hard and soft segments. Tensile analysis revealed high strain at break for all segmented copolymers between 340 and 770%, and tunable modulus between 0.76 and 29.5 MPa. Dielectric spectroscopy revealed that the composition containing 20 wt% hard segment offered the highest permittivity at 10.6 (1 kHz, 300 K) of the segmented copolymers, indicating potential as a dielectric elastomer.

## Abstract 2

Polyurea elastomers derived in part from a bio-sourced feedstock and synthesized using an isocyanate-, solvent-, and catalyst-free approach exhibit elastomeric properties while maintaining melt-processibility.

## Graphical abstract

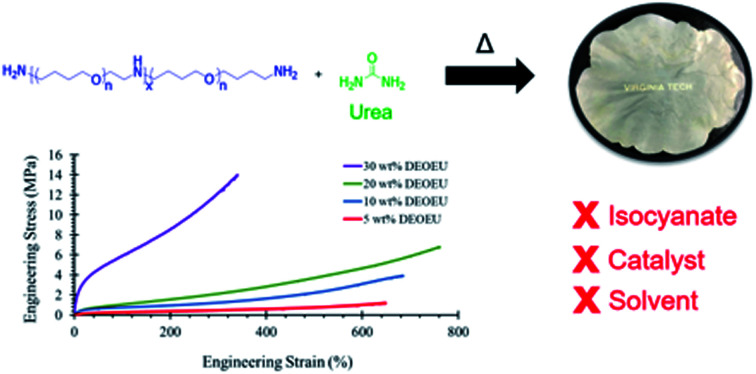

## 1. Introduction

Thermoplastic polyurea elastomers (TPUr) consist of alternating, covalently linked soft segments and hard segments. A low *T*_g_, flexible polymer defines the soft segments and provides flexibility to the copolymer while the hard segments act as physical crosslinks and impart mechanical strength. The most commonly employed soft segment oligomers in TPUr include amine-terminated poly(ethylene oxide) (PEO),^1^ poly(propylene oxide) (PPO),^2^ poly(tetramethylene oxide) (PTMO),^3–5^ and poly(dimethyl siloxane) (PDMS).^6,7^ Typically, the hard segment forms from the reaction of the soft segment diamine with a diisocyanate and an optional small-molecule diamine chain extender. The resulting urea linkages in the hard segment form strong bidentate hydrogen bonds between polymer chains. The elastomeric properties of TPUr arise from microphase separation that occurs between the hard and soft segments. The degree of microphase separation largely depends on the solubility parameters between the urea units and the chosen soft segment as well as the segment length and the Flory–Huggins interaction parameter (*χ*).^8^ Segmented polyureas generally exhibit better phase separation than polyurethanes owing to the increased polarity of the urea linkage and propensity for bidentate hydrogen bonding, and thus benefit from superior mechanical properties.^9^ Many microphase separated TPUr exhibit high tensile strains at break (*i.e.* ≫100%) while also achieving large ultimate stresses (*e.g.* >15 MPa) due to the strength of the hydrogen bonding.^3,9,10^

As mentioned previously, the synthesis of polyureas classically involves the reaction of highly toxic diisocyanates with diamines, which poses significant human and environmental health concerns. In addition, toxic catalysts and volatile organic solvents are also frequently employed to facilitate the reaction. In response to these hazards, several isocyanate-free routes to polyureas exist in the literature. The direct incorporation of CO_2_ with amines leveraging ionic liquids as a green catalyst affords polyureas; however, this method still requires the use of toxic solvents and high pressures, which limit its application.^11^ Transurethanization between a diamine and a biscarbamate provides an alternate route towards isocyanate-free polyureas, but requires one or more additional synthetic steps before polycondensation.^12^ Furthermore, this synthetic route still requires the use of organic solvents and catalysts. Leibler *et al.* previously demonstrated the ability to synthesize polyurea networks from the melt polycondensation of urea and multifunctional amine-derivatized fatty acids in the absence of a catalyst.^13,14^ This reaction utilized urea as a non-toxic, biologically derived, and relatively inexpensive substitute for isocyanates. Recently, our group also utilized this reaction to synthesize a series of semicrystalline, thermoplastic polyurea copolymers with tunable crystalline melting points.^15^ Sirrine and Long *et al.* further expanded on this approach for the synthesis of segmented PDMS-based TPUr with various hard segment contents.^16^ The TPUr exhibited high strain at break between 495 and 1180% dependent on hard segment content. However, the hard segment incorporation for these materials did not exceed 4 wt%, and thus the maximum stress at break did not exceed 1.16 MPa.

Polymers that exhibit high relative permittivity, or dielectric constant, find use in energy storage devices such as capacitors and actuators.^17,18^ Currently, polysiloxane and acrylic elastomers comprise much of the literature regarding dielectric elastomer actuators (DEA); however, these materials exhibit low relative permittivity values ranging from 3–4. Recent literature details methods for increasing permittivity of these materials through covalent attachment of dipolar substituents or blending with high permittivity fillers, but these modifications generally result in a corresponding increase in the Young's modulus, which is detrimental to DEA performance. Similarly, biaxially oriented polypropylene (BOPP) remains the benchmark material for high energy density capacitors owing to its high breakdown strength, low dielectric loss, and ease of processability despite having a low dielectric constant (∼2.2 at room temperature).^19^ Polyureas and polyurethanes have an intrinsically higher dielectric permittivity than silicone and acrylic elastomers or BOPP (>7) due to the polar nature of the urea/urethane linkage.^20^ Furthermore, Lorenzini *et al.* demonstrated the ability to tune the dielectric permittivity of polyureas and polyurethanes through the incorporation of ether linkages into the polymer backbone.^21^ Through careful synthetic design, polyureas and polyurethanes with both high permittivity and low modulus are achievable.

This report describes a strategy for utilizing urea as a comonomer to form PTMO-based TPUr with hard segment contents ranging from 5 to 30 wt%. The melt polycondensation of a commercial PTMO-based diamine with urea and an ether-containing small molecule diamine in the absence of catalyst afforded a library of melt processible segmented polyureas. Thermogravimetric analysis (TGA) further confirmed the hard and soft segment content based on reaction stoichiometry while DSC revealed the thermal transitions in each polyurea. Dynamic mechanical analysis (DMA) suggested the presence of microphase separation. Tensile testing revealed high strain at break and tunable moduli for the segmented polyureas comparable to literature values for isocyanate-based polyureas. Finally, broadband dielectric spectroscopy (BDS) revealed a high dielectric permittivity and relatively low loss for the sample containing 20 wt% hard segment, indicating the potential for this composition to act as a dielectric elastomer (DE).

## 2. Materials and methods

### 2.1 Materials

2,2-(Ethylenedioxy)bis(ethylamine) (EBA) (98%) and urea (BioReagent) (≥99%) were purchased from Sigma Aldrich and used as received. Jeffamine® THF-170 was generously provided by the Huntsman Corporation. All reagents were dried overnight at 60 °C at reduced pressure to remove water before use.

### 2.2 Synthesis of poly(tetramethylene oxide urea)-*co*-poly(di(ethylene oxide)ethylene urea)s [poly(PTMOU)-*co*-poly(DEOEU)s]

The synthesis of the segmented and non-segmented polyureas follows the identical isocyanate-, solvent-, and catalyst-free melt polycondensation procedure as described in previous reports.^15,16^ The reactant amounts were calculated based on the desired hard segment content as described in detail by Sirrine and Long *et al.*^16^ The amount of EBA was calculated such that there was a 1.5 mol eq. relative to urea. In a typical synthesis for a polyurea containing 30 wt% of hard segment, Jeffamine® THF-170 (1700 g mol^−1^, 16.50 g, 9.710 mmol), urea (3.172 g, 52.81 mmol), and EBA (9.580 g, 64.65 mmol) were added to a 100 mL, 1-necked, round-bottomed flask. The flask was equipped with a custom-made t-necked glass adapter with a spherical ball joint, nitrogen inlet, and spherical socket joint. The ball joint from the t-neck was connected to a glass condensing tube with a nitrogen outlet and a corresponding spherical socket joint, which was connected to a 50 mL round-bottomed collection flask. The collection flask was cooled in a bath of dry ice and isopropyl alcohol. The glass t-neck adapter allowed an overhead metal stir rod to pass through and provide mechanical stirring, which was connected by a spherical ball joint attached to Tygon® tubing. Tygon® tubing was attached to the nitrogen outlet and fed into a bubbler of 1 M HCl solution to quench any urea that was not condensed in the cold bath. Three alternating vacuum and N_2_ purge cycles ensured that oxygen was completely removed and provided an inert atmosphere for the melt polymerization. The reaction vessel was heated under a constant flow of N_2_ (∼10 mL min^−1^) to 170 °C and stirred (∼80 rpm) for 1 h to provide a homogeneous melt before increasing the temperature. The presence of ammonia gas was observed within the first 1 h of the reaction. Subsequently, the reaction mixture was heated to 200 °C for 1 h and 220 °C for 30 min while stirring under nitrogen flow. In the final step, the reaction mixture was heated to 250 °C and vacuum was applied for 2 h to remove the excess diamine generated through transureaization. The melt viscosity increased substantially during this step resulting in the polymer wrapping around the stir rod. Non-segmented poly(PTMOU) was synthesized with an identical synthetic method without incorporating EBA as a chain extender; 1 mol eq. of Jeffamine® to urea was utilized for the non-segmented synthesis. The resulting polyureas were isolated and used without further purification.

### 2.3 Analytical methods

The PTMO-based polyureas were dried in a vacuum oven for 18 h at 60 °C prior to melt processing. The samples were compression molded at 180 °C between two sheets of silicone-treated PET separated by 0.5 mm thick shims to obtain free-standing, creasable films. Before each experiment, the films were dried overnight in a vacuum oven at 60 °C and allowed to slow cool for at least 2 h in a dry box (<5% RH) until utilized for analysis. Thermogravimetric analysis (TGA) was carried out on a TA Instruments Q500 TGA under nitrogen flow at a heating rate of 10 °C min^−1^ to 800 °C. Stepwise isothermal TGA experiments were performed on the same instrument at a rate of 10 °C min^−1^ under nitrogen. When the rate of weight change reached 1% min^−1^, the TGA was held at that temperature until the rate of change dropped below 0.1% min^−1^, at which time the temperature continued to ramp at 10 °C min^−1^. Differential scanning calorimetry (DSC) was performed using a TA Instruments Q200 DSC equipped with a liquid nitrogen cooling system. The thermal transitions were determined from the second heat cycle and were measured at a heating/cooling rate of 10 °C min^−1^ from −120 to 200 °C under a constant helium purge. Glass transition temperatures were determined from the temperature at the half-height of the endothermic step transitions, and the melting points were taken as the peak temperature of each melting endothermic event.

Dynamic mechanical analysis (DMA) was carried out using a TA Instruments Q800 DMA equipped with a liquid nitrogen gas cooling accessory. DMA experiments were performed in oscillatory tension mode at a frequency of 1 Hz and 0.1% strain such that it remained within the linear viscoelastic region with a heating/cooling rate of 3 °C min^−1^. The data collection was discontinued after the modulus dropped below 0.1 MPa or once the length of the sample, as measured by the instrument, increased more than 1%. Variable temperature Fourier transformed infrared spectroscopy (VT-FTIR) experiments were performed on a Varian 670-IR spectrometer, which was equipped with a PIKE Technologies diamond crystal variable temperature GladiATR™ attachment. Spectra were collected from 30–160 °C at every 10 °C, and every other temperature was plotted to show trends. Tensile tests and hysteresis were performed with an Instron® 5500R. Dogbone specimens were punched from films using an ASTM D638-V cutting die. Tensile tests were carried out at crosshead separation speed of 5 mm min^−1^. The single depicted stress/strain curve and the corresponding tensile values for each sample were reported as an average of 5 runs. Hysteresis experiments were conducted at a maximum of 200% strain for each sample. The strain rate was 19% min^−1^ for 5 cycles with a 10 minute hold at 0% strain between each cycle. The area under the curves was calculated using the trapezoid method to give % hysteresis.

For dielectric studies, polymer samples were hot-pressed in nitrogen ambience at 400 K using a Specac Mini-Film Maker to obtain 100 μm thick films. The films were then sandwiched between 20 mm stainless steel electrodes in a parallel-plate configuration with 100 μm silica rod spacers incorporated to maintain sample thickness. All dielectric measurements were carried out on a high resolution Novocontrol Alpha Analyzer (frequency range 10^−1^ to 10^7^ Hz) and the temperature control regulated by a QUATRO system (Novocontrol) using a jet of dry nitrogen, thereby ensuring relative and absolute errors better than 0.1 and 2 K, respectively. Before substantive measurements, the films were annealed at 400 K for at least 7 h to remove any possible adsorbed water. Selected permittivity data was taken at 300 K.

## 3. Results and discussion

**Scheme 1:**
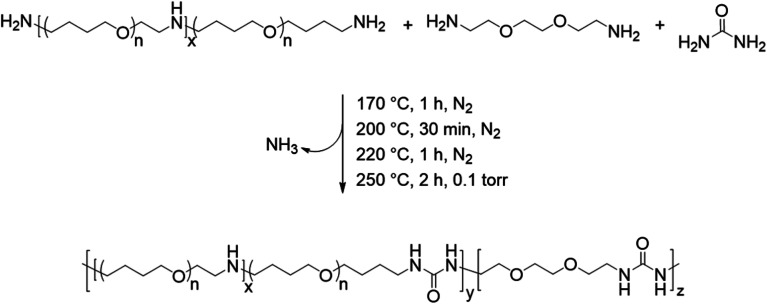
Isocyanate-free synthesis of segmented polyureas utilizing melt polycondensation.

The melt condensation of amines with urea to form substituted ureas through the *in situ* formation of isocyanic acid has been described previously in the literature.^13,15,16,22^Scheme 1 shows the unprecedented synthesis of segmented copolyureas through the melt polycondensation of a commercial PTMO-based diamine, urea, and an optional small-molecule diamine chain extender (EBA). The reaction mixture remained heterogeneous below the melting point of urea (135 °C). The urea decomposed into isocyanic acid and ammonia above 150 °C, which allowed for the reaction with the primary amines to form the 1,3-dialkyl urea linkage. A 1.5 molar excess of the EBA chain extender to urea for the segmented copolyureas helped to account for volatilization of the small-molecule EBA at the beginning of the reaction. Additionally, a stoichiometric excess of amine over urea and heating to temperatures in excess of 200 °C limited the formation of side products such as 1,1-dialkylurea, biurets, and imidazolidone cyclics.^13,16,22^ Initial reaction temperatures of 170 °C facilitated oligomerization and allowed for limited volatilization of the small molecule diamine in the early stages of the reaction. Increasing the temperature incrementally to 250 °C ensured a low melt viscosity while further facilitating the reaction. The reaction proceeded under reduced pressure at 250 °C in the final stage allowing for the removal of excess EBA and the gaseous ammonia by-product. This shifted the stoichiometry towards unity and drove the reaction to completion and high molecular weight polymer. The viscosity of the polyureas increased significantly during the vacuum step resulting in high molecular weight polymers that wrapped the mechanical stir rod. Limiting the hard segment content to 30 wt% or less ensured that the polyureas retained elastomeric properties and did not experience phase inversion resulting in a thermoplastic. Polyurea controls containing 0 wt% and 100 wt% of the hard segment were also synthesized for comparative purposes.

**Fig. 1:**
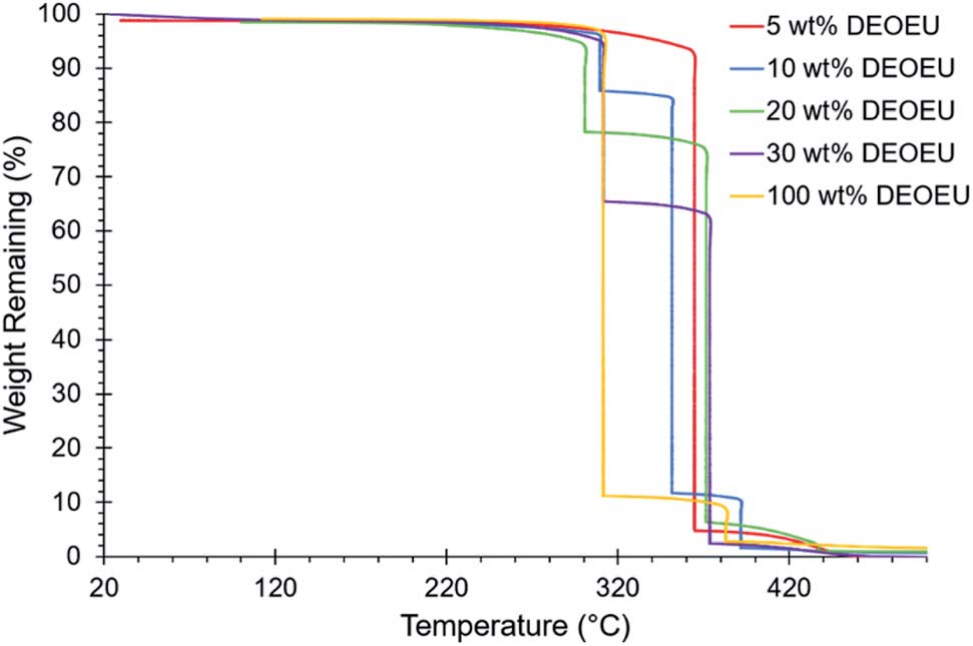
Stepwise isothermal TGA revealing the hard segment composition of the segmented polyureas.

The polyurea containing 0 wt% hard segment dissolved in common organic solvents such as chloroform and THF. Incorporation of the DEOEU hard segment rendered the segmented polyureas insoluble in most solvents due to the strongly hydrogen bonded and semi-crystalline nature of the hard segment. However, all the polyureas containing DEOEU dissolved in DMF when heated above the hard segment melting temperature of 130 °C, indicating that the polyureas were not covalently crosslinked. Lack of solubility in common NMR and SEC solvents prevented molecular weight and structural determination. However, stepwise isothermal TGA provided a method for estimating the composition of each polyurea. Segmented polyurethanes and polyureas commonly exhibit a two-step weight loss degradation profile in TGA with the hard segment degrading in the first step.^23^ As shown in Fig. 1, the weight loss at each step in stepwise isothermal TGA correlated well with the targeted hard and soft segment compositions for each segmented copolyurea. Although the 5 wt% hard segment sample did not show a sharp transition for the degradation of the hard segment as in the other segmented polyureas, the weight of the sample did decrease gradually by 6% before the soft segment degradation occurred at 365 °C. Table 1 displays the calculated weight loss for each degradation step of each polyurea. For simplicity, the targeted hard segment compositions are used herein to identify the polyureas instead of the measured TGA values.

**Table 1:** Summary of thermal properties for PTMO-based, segmented polyureas

DEOEU content (wt%)	TGA^a^					DSC^b^					DMA^c^
	*T*_d,5%_ d (°C)	*T*_d,1_ (°C)	Wt loss (%)	*T*_d,2_ (°C)	Wt loss (%)	*T*_g_ (°C)	*T*_m,1_ (°C)	*T*_m,2_ (°C)	*T*_m,3_ (°C)	Δ*H*_m,1_ (J g^−1^)	*T*_g_ (°C)
0	384	—	—	—	—	−76	23	—	—	45.5	−56
5	352	—	6	365	94	−76	15	—	—	36.0	−53
10	335	316	12	351	88	−76	12	—	—	28.9	−50
20	323	300	20	371	80	−76	14	92	129	20.7	−60
30	326	311	34	373	66	−76	10	94	129	6.23	−56
100	311	311	90	383	10	21	123^e^, 132	139	—	—	26

a Stepwise isothermal TGA, 10 °C min^−1^, N_2_.

b Second heating cycle, −120 to 180 °C, 10 °C min^−1^, He.

c Oscillatory tension mode, 1 Hz, 0.1% strain, 3 °C min^−1^.

d Temperature ramp, 10 °C min^−1^, N_2_.

e Determined from first heat.

**Fig. 2:**
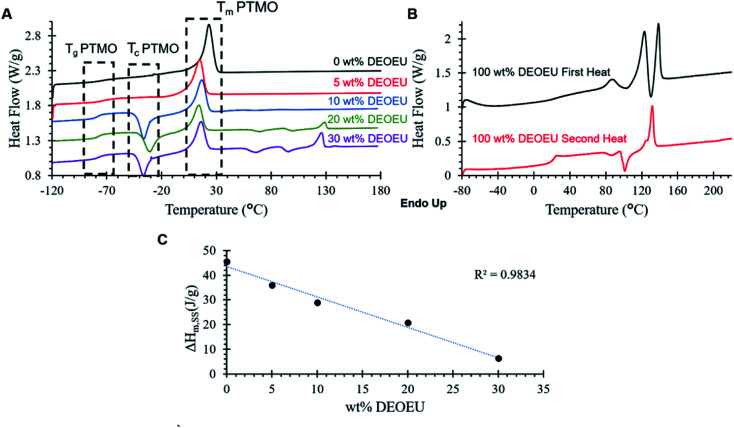
(A) DSC 2^nd^ heating scans for segmented polyureas containing various amounts of DEOEU hard segment. The curves are shifted vertically for clarity. (B) 1^st^ and 2^nd^ heating scans for DEOEU homopolymer and (C) Δ*H*_m_ for the soft segment melting endotherm as a function of hard segment incorporation.

Differential scanning calorimetry (DSC) revealed the thermal transitions of the polyureas as shown in Fig. 2. All PTMO-containing polyureas displayed a characteristic *T*_g_ at −76 °C for the soft segment. The PTMO-based soft segment displayed a melting transition centered at 23 °C, which was consistent with the endothermic transition in the 0 wt% DEOEU thermogram (Fig. 2A). Incorporation of DEOEU hard segment into the polyureas depressed the soft segment melting point to 10–15 °C as the concentration of the soft segment domains decreased. For polyureas that contained 10 wt% DEOEU incorporation or higher, cold crystallization of the soft segment occurred around −40 to −30 °C upon the second heating. At these compositions, the hard segment presumably provided sufficient physical crosslinking to restrict the mobility of the soft segment, which inhibited the ability of the PTMO to fully crystallize during the cooling step. Upon heating above the *T*_g_, the polymer chains had sufficient mobility to continue crystallizing resulting in cold crystallization (*T*_c_). A microphase separated polyurea typically displays two distinct *T*_g_s; however, in this case, the *T*_g_ for the DEOEU homopolymer (100 wt% DEOEU) was 21 °C (Fig. 2B), which overlapped with the soft segment melting endotherm and was not distinguishable. The melting enthalpy of the soft segment endotherm decreased linearly with increasing hard segment incorporation (Fig. 2C) due to the decreasing concentration of soft segment domains. This trend further suggested an absence of branching in the soft segment; branching would significantly decrease the level of crystallinity in the soft segment, which would result in a non-linear trend in the melting enthalpy.^24^

Incorporation of 20 and 30 wt% of DEOEU gave rise to two additional endothermic transitions above the soft segment melting point centered around 93 and 129 °C. The peak at 129 °C correlated with the melting point in the second heat of the 100 wt% DEOEU, and agreed with the melting point measured by Dennis *et al.*^15^ The broad peak at 93 °C coincided with the peak near the same temperature in the first heat of the 100 wt% DEOEU polyurea. This peak may indicate the presence of a polymorphic crystalline structure that arises from the DEOEU hard segment. The presence of two distinct melting transitions in the 100 wt% DEOEU polyurea seems to support this interpretation; however, further morphological characterization is required to definitively determine the crystal structure of these polyureas.

**Fig. 3:**
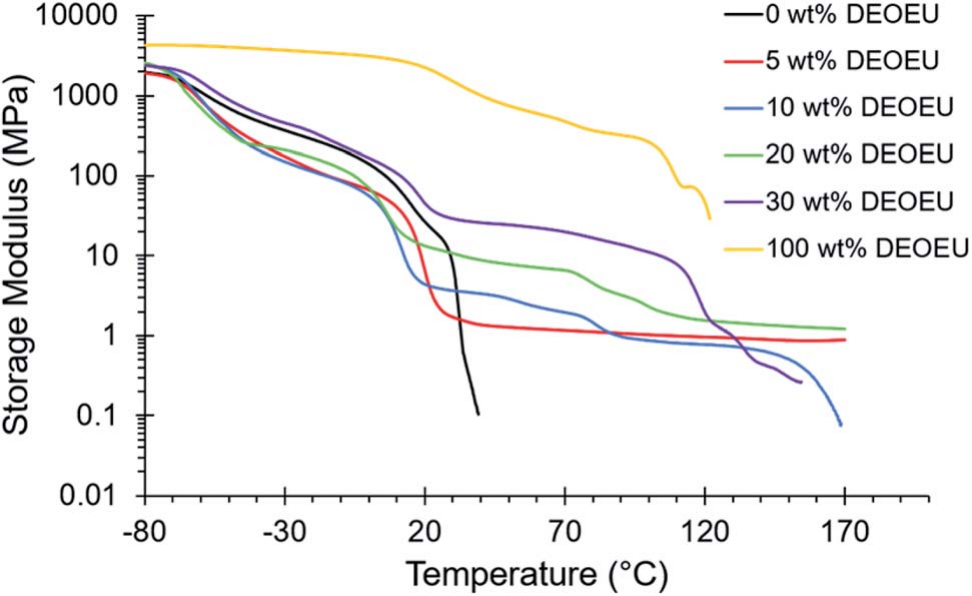
DMA heating trace of segmented polyurea copolymers.

Dynamic mechanical analysis (DMA) revealed the storage modulus as a function of temperature between −80 and 200 °C (Fig. 3). As expected, all polymers exhibited typical glassy moduli of 1–3 GPa at −80 °C. The *T*_g_ for each polyurea occurred between −60 and −50 °C as indicated by a drop in the storage modulus and a broad peak in the tan *δ* (Fig. S1†). Incorporation of hard segment resulted in further broadening of the tan *δ* peak, and in some cases (*e.g.* 30 wt% DEOEU), induced the appearance of a second peak at higher temperatures. This behavior suggested the presence of phase mixing, which is common among low molecular weight, ether-based soft segments.^9,25^ The soft segment melts between 10 to 20 °C resulting in an expected modulus drop. The presence of a plateau modulus after the PTMOU melting transition in the segmented copolyureas suggested microphase separation. The sample containing 0 wt% hard segment experienced flow soon after the melting point, whereas the hydrogen bonding and crystallinity of the hard segment in the remaining samples facilitated plateaus in the moduli after soft segment melting. As expected, the plateau modulus increased as a function of increasing hard segment content (ranging from 1 to 100 MPa) due to increased hydrogen bonding and crystallinity.^26^ A slight decrease in the plateau moduli near 80 °C corresponded to the endothermic transition shown in DSC near the same temperature for the higher hard segment containing polyureas. Above 100 °C, the hard segment begins to melt as apparent from a second peak in the tan *δ*; however, the polyureas remain physically crosslinked until 150 °C where the hydrogen bonds sufficiently dissociated to allow for flow. Variable temperature FTIR confirmed the presence of bidentate hydrogen bonding in the hard segment, and the reduction of hydrogen bonding occurred at temperatures that were consistent with the thermal transitions observed both in DSC and DMA results (Fig. S3 and S4†). Table 1 summarizes the thermal properties of the polyureas.

**Fig. 4:**
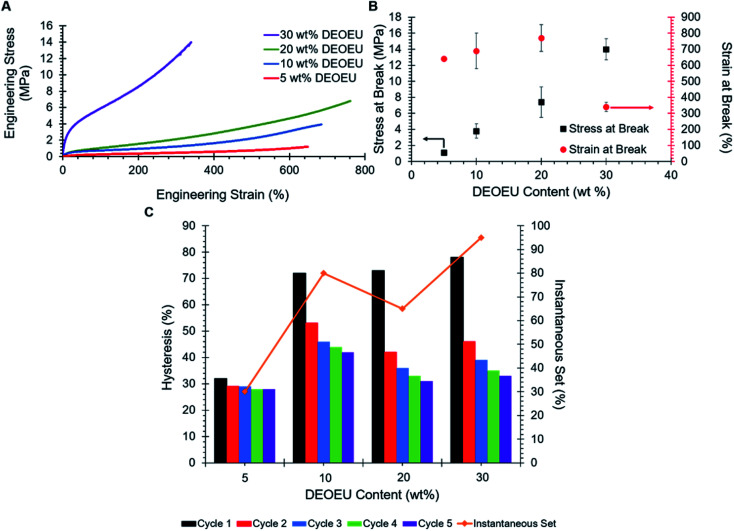
(A) Engineering stress *vs.* strain for segmented polyureas (B) stress and strain at break as a function of hard segment content and (C) five-cycle mechanical hysteresis and instantaneous set for segmented polyureas.

Fig. 4A reveals the stress–strain behavior of the segmented, PTMO-based, polyureas. Compositions consisting of 20 wt% DEOEU hard segment or less exhibited strains at break between 640 to 770%, consistent with or superior to isocyanate-based, segmented, polyureas and polyurethanes in the earlier literature.^3,9,16,27^ The ultimate stress increased systematically with increasing hard segment (Fig. 4B) from 1 to 15 MPa as the amount of physical crosslinking increased. Increasing the hard segment content to 30 wt% resulted in a significant decrease in the strain at break down to 340% as the ultimate stress increased, which was consistent with earlier literature examples.^16,27^ The mean Young's modulus also expectedly increased with increasing physical crosslinking from 0.76 MPa for 5 wt% DEOEU to 29.5 MPa for 30 wt% DEOEU (Table 2). The polyurea containing 0 wt% hard segment melted and flowed upon handling at room temperature, and the determination of mechanical properties was impossible.

**Table 2:** Summary of tensile properties for PTMO-based, segmented polyureas

DEOEU content (wt%)	Modulus (MPa)	Ultimate stress (MPa)	Strain at break (%)	Mechanical hysteresis (%)					Instantaneous set (%)
				Cycle 1	Cycle 2	Cycle 3	Cycle 4	Cycle 5	
5	0.76 ± 0.16	1.1 ± 0.10	640 ± 11	32	29	29	28	28	30
10	4.35 ± 0.29	3.8 ± 0.90	690 ± 110	72	53	46	44	42	80
20	4.54 ± 0.54	7.4 ± 1.9	770 ± 83	73	42	36	33	31	65
30	29.5 ± 1.13	14 ± 1.3	340 ± 29	78	46	39	35	33	95

Tensile hysteresis results from energy lost in the form of heat as a polymer is repeatedly stretched and released. Hysteresis in segmented polyureas partly arises from the deformation of the hard segment morphology during elongation.^9^ Straining the polymer disrupts the intermolecular hydrogen bonding in the hard segments. The polymer chains typically do not return to their original conformations upon unloading, which results in energy dissipation. Fig. 4C summarizes the five-cycle hysteresis measurements of the segmented polyureas strained to 200%. In all samples, the first cycles show the highest amount of hysteresis relative to the following cycles, as expected. This phenomenon presumably occurred due to an equilibrium morphology that was disrupted during the first loading and did not have sufficient time to reform between cycles, which resulted in the soft matrix sustaining the sequential loads.^7,28^ Consequently, the hysteresis increased with amount of hard segment in the polyurea.^7,29^ The first-cycle hysteresis increased substantially from the 5 wt% DEOEU to the 10 wt% DEOEU polyurea, and more subtly between the higher hard-segment-content polyureas. The instantaneous set, which is the strain where the stress reaches zero on the first hysteresis unloading curve, generally increased with increasing hard segment content as the disruption of the larger hard segment domains led to more unrecoverable energy loss. Given the elastomeric nature of the segmented polyureas and the presence of polarizable urea and ether linkages throughout the backbone, these materials may prove suitable for DE applications provided they also exhibit high relative permittivity.

**Fig. 5:**
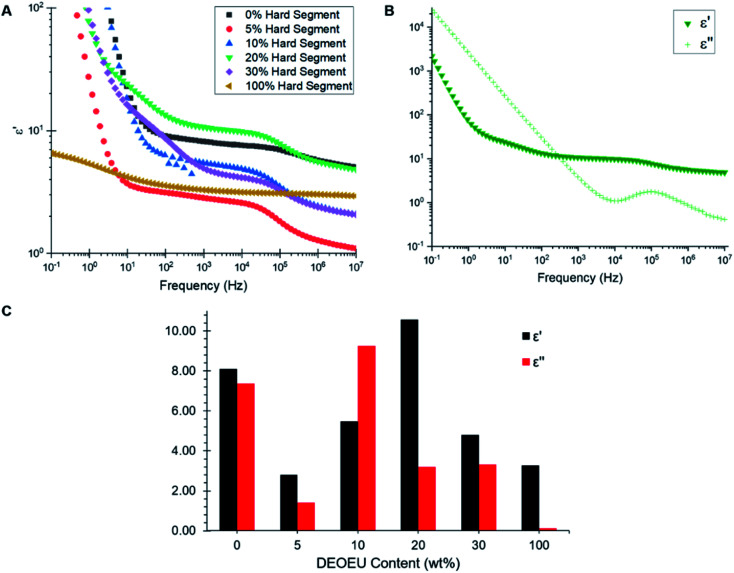
(A) Dielectric permittivity (*ε*′) as a function of frequency for the synthesized polyureas. (B) Permittivity and dielectric loss (*ε*′′) as a function of frequency for the polyurea containing 20 wt% hard segment. (C) Comparison of dielectric permittivity and dielectric loss at a frequency of 1 kHz.

Broadband dielectric spectroscopy revealed the relative permittivity and dielectric loss for each polymer. Fig. 5A shows an overlay of the dielectric permittivity as a function of frequency for each composition at 300 K. At low frequencies, the permittivity increased sharply due to conductivity contributions. Therefore, the relative permittivity values were obtained at higher frequencies (>100 Hz), which is the spectral region where minimal dispersion phenomena appear to occur in these samples. The plateaus in the dielectric permittivity spectra revealed an interesting trend. The 5 wt% DEOEU sample displayed the lowest relative permittivity with a plateau permittivity on the order of 2.8. The permittivity initially increased with increasing hard segment as expected due to an increase in the number of ether linkages present in the backbone. However, the permittivity peaked near 10.6 for the 20 wt% DEOEU sample and started to decline with further hard segment incorporation. The origin of this decrease in permittivity presumably stems from an increase in the hard segment crystallinity as the crystalline domains restrict the ability of the DEOEU linkages to polarize. DSC analysis appeared to support this hypothesis since the hard segment melting peaks did not appear until the 20 wt% DEOEU sample. However, the 20 wt% DEOEU sample displayed the highest permittivity despite also demonstrating hard segment crystallinity in the DSC. This observation suggested that the percentage of the amorphous phase remains significantly higher than the crystalline phase at this level of DEOEU incorporation. Confirming this hypothesis will require a more in-depth morphological analysis in the future.

Fig. 5B shows a plot of dielectric permittivity and loss as a function of frequency for the 20 wt% DEOEU sample as a representative example. The dielectric loss quantifies the electromagnetic energy dissipated in a dielectric material, and thus should preferably remain low for DE applications. The dielectric loss starts high at low frequencies and drops to a minimum at 10 kHz before peaking again at higher frequencies. Fig. 5C provides a visual representation of the relative permittivity and dielectric loss of the polyureas at 1 kHz, which is a relevant frequency for DE applications. Interestingly, the graph shows no apparent trend in the dielectric loss as a function of DEOEU hard segment content. Although the polyurea containing 0 wt% DEOEU has a high permittivity around 8 (Table 3), it also suffers from a high dielectric loss. Additionally, this sample lacks any significant mechanical properties at room temperature as discussed previously, which excludes its use as a DE. The 100 wt% DEOEU polymer conversely enjoys a very low dielectric loss compared to the other compositions. Despite this attribute, the permittivity remains relatively low at around 3, and this composition exhibits mechanical properties typical of a thermoplastic as shown by Dennis and Long *et al.*^15^

**Table 3:** Summary of dielectric properties for the segmented polyureas at a frequency of 1 kHz

Hard segment content (wt%)	*ε*′ (1 kHz)	*ε*′′ (1 kHz)	tan *δ*
0	8.09	7.36	1.12
5	2.81	1.41	0.50
10	5.47	9.26	1.69
20	10.6	3.20	0.30
30	4.80	3.31	0.69
100	3.27	0.13	4.00 × 10^−3^

As for the segmented copolymers, the 20 wt% DEOEU sample demonstrated the highest permittivity at 10.6. This permittivity sits significantly higher than many functionalized silicone and acrylics utilized in literature for DEs; however, this permittivity increase also corresponds with an increased Young's modulus compared to conventional DEA materials.^18^ This composition also exhibits a relatively high dielectric loss when considering its application as a dielectric material.^30^ Regardless, with more tuning, this composition may function effectively as a DEA due to its high permittivity and ability to achieve a large strain at break, and our future efforts will evaluate strategies for decreasing the dielectric loss while maintaining (or improving) the dielectric permittivity.

## 4. Conclusions

The melt polycondensation of a commercially available, PTMO-based diamine with urea and a chain extender yielded a series of melt processible polyureas synthesized in the absence of toxic isocyanates, catalysts, and solvents. Polyureas with varying hard segment contents formed free-standing creasable films upon melt compression. DSC analysis revealed multiple endothermic transitions in the segmented copolyureas correlating to the soft segment and hard segment melting points. DMA further suggested microphase separation and provided evidence for phase mixing between the hard and soft segments. Tensile analysis revealed high strain at break in the segmented polyureas between 340 to 770% strain, comparable to literature examples of isocyanate-based polyureas. Young's modulus and ultimate stress increased significantly with increasing hard segment incorporation indicating the variability of attainable mechanical properties using this method. Five-cycle hysteresis measurements revealed increasing first-cycle hysteresis with increasing DEOEU incorporation due to increasing hard segment domains. Correspondingly, the instantaneous set also increased with the DEOEU content. Broadband dielectric spectroscopy revealed that the polyurea containing 20 wt% DEOEU displayed the highest dielectric permittivity (10.6) of the segmented copolymers measured at 1 kHz and 300 K. This data coupled with the tensile properties suggests that this polyurea composition may prove suitable as a candidate for DEA applications provided that the dielectric loss is lowered upon further modification.

## Conflicts of interest

There are no conflicts of interest to declare.

## Supplementary Material

RA-010-D0RA02369H-s001
