# Supplementary material for: Isocyanate- and solvent-free synthesis of melt processible polyurea elastomers derived from urea as a monomer
Source: RSC Adv. 2020 May 18;10(32):18760–8. doi: 10.1039/d0ra02369h (PMC9054001; doi:10.1039/d0ra02369h)
Supplement: RA-010-D0RA02369H-s001 [file RA-010-D0RA02369H-s001.pdf]

Electronic Supplementary Material (ESI) for RSC Advances.
This journal is © The Royal Society of Chemistry 2020

Supporting Information

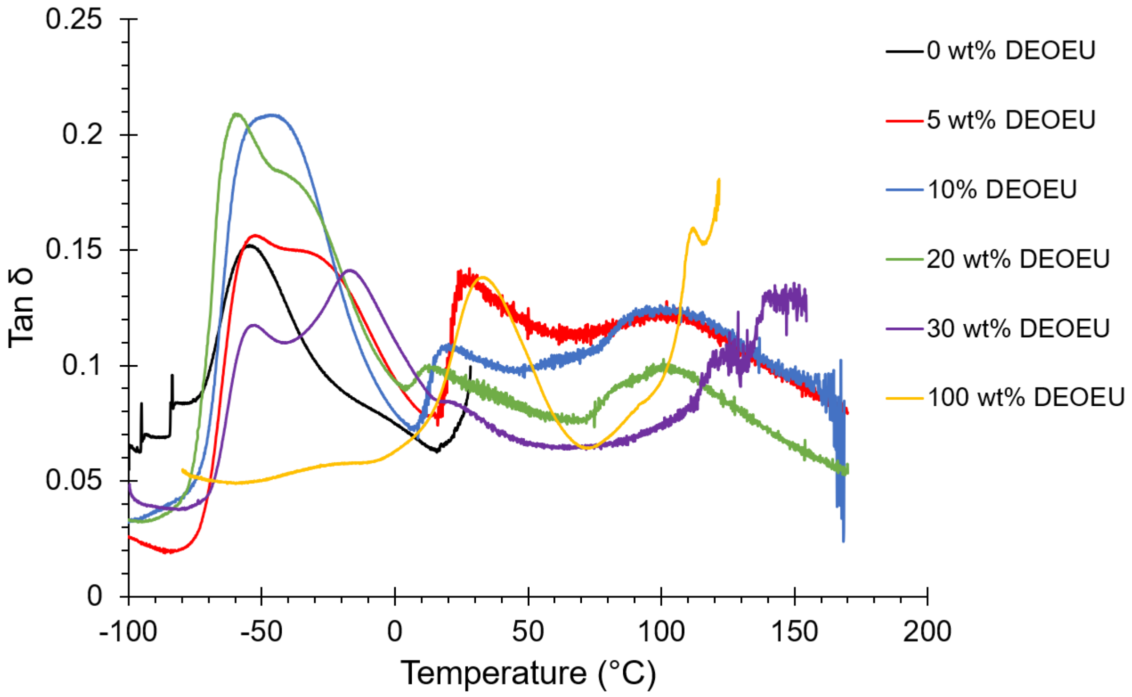

Figure S1: Corresponding DMA tan δ behavior for polyurea homopolymers and copolymers

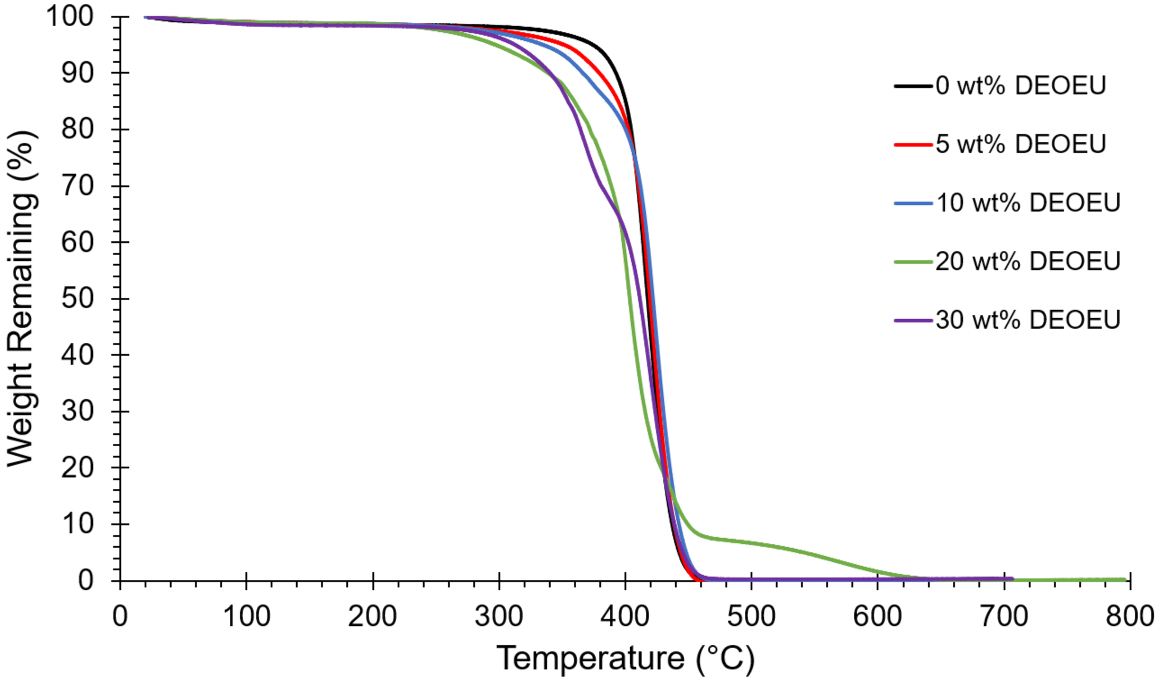

Figure S2: Thermogravimetric analysis utilizing a 10 °C min^{-1} temperature ramp to 800 °C for the series of polyureas

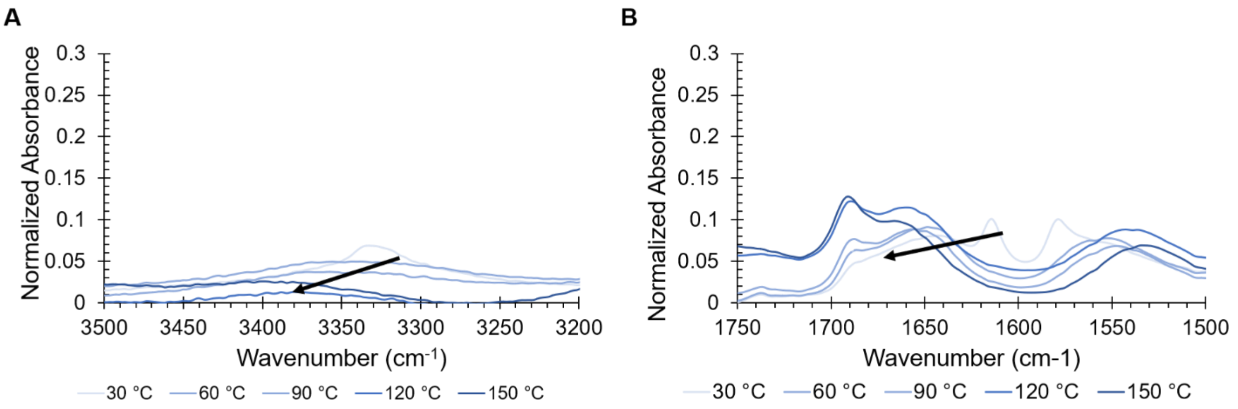

Figure S3: Variable temperature FTIR of the 0 wt% DEOEU polyurea showing (A) the N-H stretching band shifting from ~3325 cm^{-1} for strongly hydrogen bonded ureas to ~3350 cm^{-1} for weakly hydrogen bonded with increasing temperature (B) the amide band shifting from ~1650 cm^{-1} for strong hydrogen bonding to ~1690 cm^{-1} for weak hydrogen bonding with increasing temperature

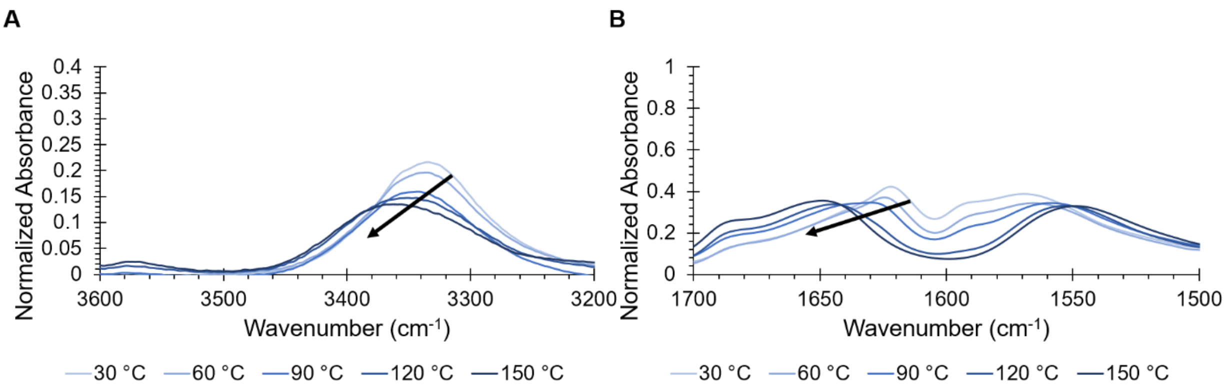

Figure S4: Variable temperature FTIR of the 30 wt% DEOEU polyurea showing (A) the N-H stretching band shifting from ~3325 cm^{-1} for strongly hydrogen bonded ureas to ~3350 cm^{-1} for weakly hydrogen bonded with increasing temperature (B) the amide band shifting from ~1650 cm^{-1} for strong hydrogen bonding to ~1690 cm^{-1} for weak hydrogen bonding with increasing temperature
